# Possible Mechanisms of mRNA-LNP Degradation: A Comprehensive Review

**DOI:** 10.3390/vaccines14070620

**Published:** 2026-07-15

**Authors:** MD Faizul Hussain Khan, Tahsina Islam, Abhishek Mishra, Amine A. Kamen

**Affiliations:** 1 Viral Vectors and Vaccines Bioprocessing Group, Department of Bioengineering, McGill University, Montreal, QC H3A 0G4, Canada; md.f.khan@mail.mcgill.ca (M.F.H.K.); abhishek.mishra@mcgill.ca (A.M.); 2 Department of Microbiology, Jahangirnagar University, Dhaka 1342, Bangladesh; tahsina013@gmail.com

**Keywords:** mRNA-LNP vaccines, mRNA degradation, lipid nanoparticle (LNP) stability, LNP degradation, phosphodiester hydrolysis, lipid oxidation, RNase contamination, physicochemical instability, freeze–thaw stress, lyophilization, cold-chain logistics, nanoparticle aggregation, nucleic acid delivery systems

## Abstract

Messenger RNA-lipid nanoparticle (mRNA-LNP)-based drug products represent a promising platform for prophylactic and therapeutic applications. However, their limited stability poses significant challenges for storage and global distribution. The instability of mRNA-LNP products makes them dependent on ultra-cold chain systems. This instability is driven by various physicochemical factors, including temperature, pH, light exposure, oxidation, aggregation, shear stress, and humidity. These factors destabilize the physical and chemical integrity of both mRNA and lipid nanoparticle (LNP) components, leading to reduced vaccine potency and potentially increasing the risk of adverse safety outcomes. Understanding these factors and their mechanisms is crucial for retaining mRNA-LNP efficacy. This review discusses the key physicochemical instability factors and molecular degradation mechanisms responsible for the structural and functional deterioration of mRNA-LNP formulations. Further, we summarize the stabilization strategies and analytical methods used to detect and quantify the degradation of mRNA-LNP products. Addressing these challenges is critical for advancing next-generation nucleic acid-based drug products and LNP-based delivery systems.

## 1. Introduction

Messenger RNA (mRNA) vaccines play an effective role in the prevention of infectious diseases. They are considered an innovative approach for immunization and have emerged as a promising alternative to traditional vaccines [1]. mRNA vaccines from Pfizer/BioNTech and Moderna have significantly changed the course of the SARS-CoV-2 pandemic [2,3]. Their rapid development was supported by the efficacy and safety of mRNA demonstrated in phase 3 clinical trials. Compared to traditional vaccines, mRNA vaccines offer advantages such as easy and fast antigen design, safety, low cost, and high production capacity, making them an ideal technology platform in sudden outbreaks and large-scale immunization needs [4]. Consequently, the mRNA modality has been proposed as the top priority for implementing the pandemic readiness program (CEPI, 100 Days Mission).

mRNA’s rapid biodegradability contributes to its safety because it is inherently transient and does not persist in the body, but at the same time, this instability also limits its shelf life and delivery [5]. Ensuring the stability of mRNA drug products is important for maintaining their immunogenicity across the supply chain. The World Health Organization (WHO) and United Nations Children’s Fund (UNICEF) anticipate substantial annual cost savings with the use of heat-stable vaccination modalities [6]. Current mRNA vaccines require ultra-cold storage; for example, Pfizer’s vaccine must be stored below −70 °C, while Moderna’s must be kept below −20 °C [7]. Therefore, stability remains a bottleneck for the development and delivery of mRNA vaccines.

One of the key limitations of this vaccine platform is the intrinsic fragility of mRNA molecules. Unlike DNA, which is chemically more stable, mRNA is prone to rapid degradation through chemical and enzymatic mechanisms. Environmental factors such as temperature variations, pH shifts, and mechanical stress can significantly reduce mRNA stability and efficacy [8,9]. Chemical interactions like oxidation or hydrolysis can further degrade mRNA into smaller fragments [10]. This instability highlights major challenges across all stages of mRNA production, including in vitro transcription (IVT), purification, formulation, storage, and distribution.

**Figure 1:**
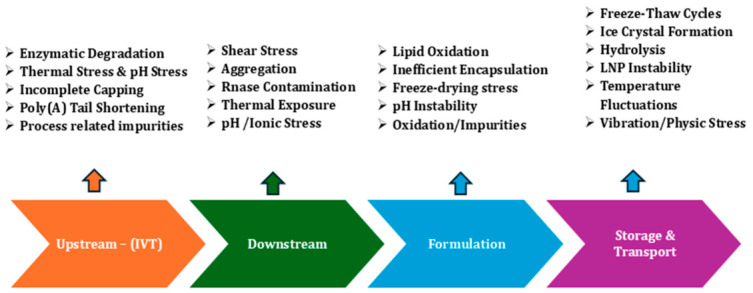
Instability factors across the mRNA-LNP workflow. Key factors affecting mRNA and LNP stability throughout manufacturing and distribution are illustrated. During IVT (upstream), risks include enzymatic degradation, thermal and pH stress, incomplete capping, and tail shortening. Downstream processing introduces shear, aggregation, RNase contamination, and pH/ionic stress. In formulation, encapsulation inefficiency, oxidation, freeze-drying, and pH instability may occur. During storage and transport, freeze–thaw cycles, hydrolysis, temperature fluctuations, and physical stress further compromise stability.

Figure 1 shows four stages of the mRNA-LNP production. Several upstream factors that originate during in vitro transcription and carry over to the downstream part. Incomplete 5′ capping leaves a fraction of transcripts vulnerable to 5′ → 3′ exonucleolytic attack and reduces translational capability. Also, shortening of the poly(A) tail removes 3′-end protection and accelerates deadenylation-dependent deterioration. Process-related impurities, including residual enzymes, template DNA, double-stranded RNA, and trace metals, can influence both enzymatic and metal-catalyzed degradation later in the process. These upstream limiting factors are discussed in Section 4.1.1 (capping and poly(A) integrity) and Section 4.4.4 (impurities), while the downstream, formulation, and storage factors are discussed in Section 4.2, Section 4.3 and Section 4.4. Section 5 and Section 6 address, respectively, the strategies used to overcome them and the analytical methods used to detect and quantify them.

Beyond the intrinsic fragility of mRNA, the lipid nanoparticle (LNP) carrier introduces additional stability limitations. Ionizable lipids, phospholipids, cholesterol, and PEGylated lipids can undergo oxidation and hydrolysis during storage [11]. These reactions can produce reactive aldehydes and other impurities that form covalent modifications called adducts with nucleosides and accelerate mRNA degradation [7,12,13]. In addition, LNP morphology and encapsulation efficiency are highly sensitive to temperature, pH, and mechanical agitation. These stress conditions can induce particle fusion, leakage of encapsulated mRNA, aggregation, or phase separation, all of which affect delivery efficiency [14,15]. Furthermore, the presence of residual water in the LNP core and in the bulk formulation facilitates hydrolytic reactions of both mRNA and ester-containing lipids [13,16,17,18]. A thorough understanding of vaccine degradation is therefore essential for mRNA-LNP formulation to identify degradation pathways and establish in-process analytical protocols. These studies not only support regulatory compliance but also enable the development of robust formulations and optimized proper storage conditions [19]. Strategies such as optimization of ionizable lipid structure, excipients, buffer pH and composition, antioxidants, and development of robust lyophilized LNP formulations with suitable cryo- and lyoprotectants are therefore central to improving LNP stability and enabling extended storage under less stringent conditions [20].

This review will comprehensively explore the various degradation mechanisms across the entire process of mRNA-LNP vaccine manufacturing, storage, transportation, and delivery. It will also highlight the potential factors contributing to the instability of this platform. Further, the review will contribute to explaining how these factors affect the stability of the mRNA-based vaccine, eventually compromising its safety and efficacy. This review also aims to summarize the stabilization strategies developed to mitigate mRNA-LNP instability and the analytical methods used to detect and characterize their degradation.

## 2. Key Component of the Drug Substance: mRNA

The drug-substance mRNA is engineered to resemble a mature eukaryotic transcript while maximizing stability and translational efficiency. There are some key structural features that directly influence degradation. The 5′ cap (e.g., m7GpppN with 2′-O-methylation) is the single most important determinant of 5′-end stability. It blocks 5′ → 3′ exonucleases and innate immune sensing, so incomplete or hydrolyzed caps are a primary degradation liability [21]. The poly(A) tail governs 3′-end stability in a length-dependent manner. Tails below ~50 nt give insufficient protection, whereas tails beyond ~150 nt can impair ribosome loading, making tail length a tunable stability parameter [9]. The 5′ untranslated region (UTR) optimizes ribosome recruitment and enhances stability [22]. UTR secondary structure and GC content modulate susceptibility to in-line hydrolysis, since AU-rich, single-stranded regions are cleaved fastest (Section 4.1.2). Modified nucleosides such as N1-methylpseudouridine improve translation and reduce immunogenicity but do not, by themselves, confer chemical stability against hydrolysis [23]. These features, rather than the coding sequence per se, are the levers available to formulators and sequence designers for improving mRNA stability.

## 3. Key Component of the Delivery Vehicle: Lipid Nanoparticles (LNPs)

**Figure 2:**
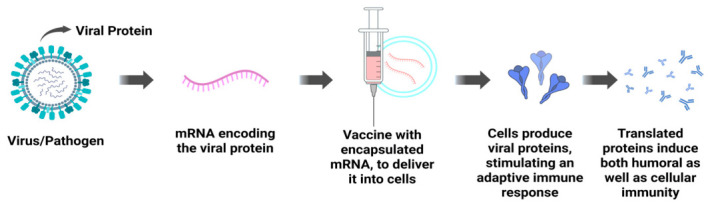
Schematic of mRNA vaccine design of antigen and mechanism of action. mRNA encoding a selected viral antigen is synthesized and encapsulated in a delivery system (e.g., lipid nanoparticles) for cellular uptake. Following administration, host cells translate the mRNA into the target antigen, which is presented via MHC-I and MHC-II pathways to prime both humoral (antibody) and cellular (CD8^+^ and CD4^+^ T-cell) immunity against the pathogen.

For effective delivery and protection, the engineered mRNA is encapsulated within a lipid nanoparticle (LNP) delivery system (Figure 2). The size of the LNP can be around 60–120 nm. The mRNA-LNPs used in vaccines (e.g., COVID-19 products) typically consist of 4-component systems, including an ionizable cationic lipid, a phospholipid (often DSPC or DOPE), cholesterol, and a PEGylated lipid at molar ratios roughly in the range of 40–50%:10–15%:35–45%:1–3%, respectively [24]. These molar ratios (ionizable lipid 40–50%, phospholipid 10–15%, cholesterol 35–45%, and PEG-lipid 1–3%) are representative of the licensed COVID-19 products [25,26]. The ionizable cationic lipids (e.g., ALC-0315 in the Pfizer-BioNTech vaccine) bind and encapsulate mRNA at low pH and become neutral at physiological pH to facilitate endosomal escape [25]. The helper phospholipids, such as DSPC, regulate the membrane’s packing density and fusion efficiency [25,26]. Cholesterol is included to enhance membrane fluidity and particle integrity to stabilize the nanoparticle [27]. Lastly, the PEGylated lipids (e.g., PEG-DMG) provide steric stabilization and prolong systemic circulation [27]. Together, these components protect mRNA, enable cellular uptake, and facilitate endosomal escape.

## 4. mRNA-LNP Vaccine Degradation Mechanisms

**Figure 3:**
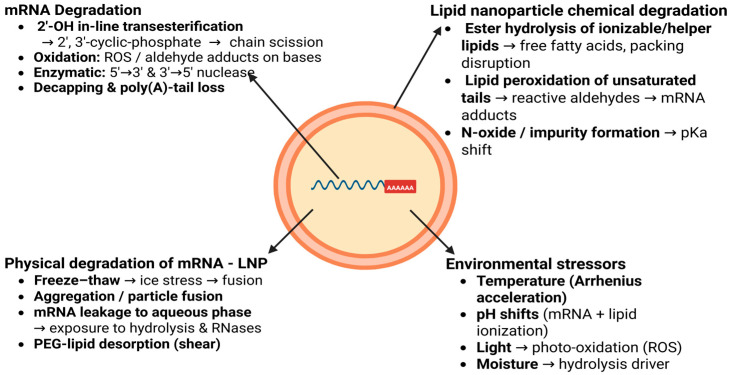
Major degradation pathways of mRNA-LNP formulations. The schematic summarizes chemical and physical instability mechanisms, including mRNA backbone hydrolysis, oxidation, enzymatic degradation, poly(A) tail loss, lipid hydrolysis and oxidation, as well as aggregation, leakage, structural changes, and freeze–thaw damage. These processes are influenced by environmental stressors such as temperature, pH, light, and moisture.

Degradation of mRNA-LNP involves two separate pathways, including degradation of the mRNA itself and degradation of the lipid nanoparticle (LNP) delivery system (Figure 3). These two processes are highly interdependent, as instability in one component can accelerate degradation of the other, ultimately reducing antigen expression and immunogenicity [28].

### 4.1. mRNA Degradation Mechanisms

#### 4.1.1. Structural/Functional Elements

Multiple structural and sequence elements contribute to mRNA instability and function. These include the 5′ cap structure, the poly(A) tail associated with poly(A)-binding protein (PABP), intramolecular secondary structures, start and stop codons, and regulatory sequences within the untranslated regions (UTRs). Together, these features help protect the transcript from nuclease attack and modulate its molecular interactions [29]. The stability of mRNA is strongly influenced by the length and composition of the poly(A) tail. In particular, tails shorter than approximately 50 nucleotides provide limited protection at the 3′ end, making the RNA more susceptible to exonucleolytic degradation. Long poly(A) tails (>150 nucleotides) can make RNA more resistant to degradation, but they can also prevent binding to ribosomes, thereby reducing the efficiency of RNA translation [30,31]. Further, increasing deadenylation allows de-capping and exposes the transcript to 5′ → 3′ exonucleases like Xrn1 and 3′ → 5′ degradation by the exosome. This disruption of stabilizing UTR elements or RNA structures [30] increases accessibility to endonucleases [32]. In parallel, loss or misplacement of functional coding-region elements (e.g., normal stop codon, reading frame integrity) activates degradation pathways, which use endonucleolytic cleavage together with deadenylation, de-capping, and exosome recruitment to rapidly eliminate defective or structurally impaired mRNAs [29]. Misfolding or loss of higher-order structure reduces ribosome payload and increases nuclease access, further promoting degradation [4].

#### 4.1.2. Hydrolysis of the Phosphodiester Backbone

Non-enzymatic degradation of mRNA primarily proceeds through hydrolytic phosphodiester backbone cleavage, often catalyzed by metal ion complexes [33,34]. In the metal-catalyzed non-enzymatic route, the ribose 2′-hydroxyl group is deprotonated and acts as an internal nucleophile that attacks the adjacent phosphorus atom. This ultimately forms a transient 2′,3′-cyclic phosphate intermediate which subsequently hydrolyzes to yield fragmented and non-translatable RNA with 2′- and 3′-phosphate termini. Under neutral to mildly alkaline conditions, this intramolecular transesterification follows apparent Arrhenius-type first-order kinetics [4,29,35]. This reaction process increases sharply with temperature, hydroxide ion activity, and the presence of divalent cations such as Mg^2+^, Mn^2+^, or Pb^2+^. This process can also lower the activation barrier by positioning and activating the 2′-OH in the pentacoordinate transition state [4,36]. Further, AU-rich segments are cleaved faster than GC-rich base-paired helices. The main reason behind this cleavage is base stacking and hydrogen bonding. Consequently, mRNA constructs with extensive unstructured regions or long AU-rich segments are intrinsically more prone to hydrolysis. Therefore, rational design strategies are often required to minimize such motifs through optimized codon usage. This engineered secondary structure of mRNA is required to enhance overall physicochemical stability [33,36,37,38].

#### 4.1.3. Oxidation and Other Chemical Degradations

mRNA-LNP is also particularly sensitive to oxidation by reactive oxygen species generated during manufacturing, storage, or exposure to light and trace metals. Oxidative degradation of the ionizable and helper lipids produces lipid peroxides and downstream aldehydes that can diffuse into the encapsulated mRNA and react with nucleobases and the ribose. This process gives rise to base lesions (for example, 8-oxo purine adducts), strand breaks, and altered secondary structure that together obstruct ribosomal recognition [39,40,41,42]. In parallel, direct oxidation of the RNA by dissolved ROS or trace transition metals can promote similar nucleobase modifications and backbone scission. Oxidation can also be triggered by atmospheric pollutants such as ozone, which reacts rapidly with the mRNA [36,39,43,44]. Packer et al. demonstrated cytosine adduct formation by LC-MS and linked it directly to loss of translational activity [45]. In contrast, direct oxidation of nucleobases by dissolved ROS or trace metals is observed in RNA/DNA radiation and oxidation-chemistry studies [46,47]. This is largely inferred for formulated mRNA–LNP rather than directly demonstrated in-product.

#### 4.1.4. Enzymatic Degradation

Protein RNases and catalytic ribozymes can also degrade mRNA either exonucleolytically, by trimming nucleotides from the transcript ends (5′–3′ exonucleases acting after cap removal and 3′–5′ exosome complexes acting after deadenylation), or endonucleolytically, by introducing internal cuts that fragment the strand [4,48]. Within cells, mRNA degradation occurs through well-established pathways in which shortening of the poly(A) tail is typically followed by removal of the 5′ cap and subsequent exonucleolytic digestion. In the case of therapeutic or vaccine mRNA, degradation can be further intensified by exogenous RNase contamination introduced during manufacturing, formulation, or handling. Consequently, even small fractions of leaked or inadequately protected mRNA may be rapidly degraded before they can access the cytosolic translational machinery [32,49,50].

### 4.2. mRNA Degradation: Key Factors

The key factors influencing mRNA vaccine degradation are critical to understanding their stability and efficacy (Table 1). These factors can affect both the integrity of the mRNA and its ability to produce the desired protein upon administration.

#### 4.2.1. Temperature

mRNA vaccines are highly sensitive to thermal degradation and greatly rely on cold-chain facilities. They are inherently unstable, as exposure to elevated temperatures accelerates chemical and enzymatic degradation processes [30]. They are often stored at very low temperatures from −20 to −80 °C to minimize degradation (Table 2). Any deviation can lead to degradation. Improper freezing and thawing cycles can also affect mRNA integrity [42]. For example, the Moderna (mRNA-1273) vaccine remains stable at 2–8 °C for up to 30 days and can tolerate room temperature for a maximum of 12 h [4]. This vaccine should not be refrozen after it is thawed and needs to be diluted right before use, whereas the Pfizer-BioNTech (Comirnaty) vaccine is stable at 2–8 °C for 5 days but can only remain stable at room temperature for up to 2 h [7,8].

Temperature influences mRNA stability by accelerating chemical degradation pathways, primarily through enhanced hydrolysis of the phosphodiester backbone. This increases with rising temperature following Arrhenius kinetics, reducing the half-life of mRNA [11]. Higher temperatures also destabilize mRNA secondary structures (less negative ΔG of folding) [11,41]. Under thermal stress, encapsulated mRNA degrades up to ~9-fold more slowly than naked mRNA [51]. In mRNA-LNP vaccine formulations, elevated temperatures (e.g., 25–40 °C) promote mRNA strand scission, resulting in decreased gene expression over weeks [52]. Therefore, cold-chain or freeze-drying remains essential to preserve full-length mRNA integrity and bioactivity during storage and distribution.

**Table 2:** mRNA Vaccines with their recommended storage temperatures [4,7,45,53,54].

mRNA Vaccine	Frozen (Long-Term Storage)	Frozen Storage Duration	Refrigerated Storage (2–8 °C)	Room Temperature Storage
Comirnaty (Pfizer-BioNTech), 2025–2026	−90 to −60 °C	Until labeled expiry date when stored continuously frozen	Up to 10 weeks after thawing	Up to 12 h total, including thawing and handling
Spikevax (Moderna), 2025–2026	−50 to −15 °C	Until labeled expiry date when stored continuously frozen	Up to 60 days after thawing	8–25 °C for up to 12 h
CVnCoV (CureVac)	−60 °C	Up to 3 months (development-stage data)	Not established	Not established
BNT162b2 (early clinical formulation)	−70 °C	Up to 6 months (early formulation)	Limited stability data during development	Limited stability data

#### 4.2.2. Light Exposure

Pfizer/BioNTech and Moderna vaccines have been reported to be unstable in light. Light exposure, particularly ultraviolet and high-energy visible wavelengths, drives photo-oxidation [53,55]. Absorbed energy and photosensitized reactions generate reactive oxygen species that oxidize nucleobases [49,50]. Additional evidence supports direct damage to encapsulated mRNA. Under light exposure, eGFP-LNPs retained high encapsulation efficiency but suffered severe mRNA degradation and near-complete loss of transfection activity [56]. Another mRNA-LNP study showed decreased luciferase expression despite unchanged particle size, PDI, and mRNA retention, with intact mRNA length [57]. Therefore, the practical recommendation to store formulations in amber or opaque vials and to minimize light exposure during handling, manufacturing, and storage is precautionary [4].

#### 4.2.3. pH

The pH of the vaccine storage medium can significantly impact its integrity. mRNA molecules exhibit higher risk of hydrolytic damage, particularly at extreme pH levels in both acidic and alkaline conditions [42]. This damage can lower the effectiveness of the vaccine by reducing the amount of intact mRNA required for translation. Research also indicated that mRNA degradation can occur rapidly at elevated pH levels, particularly in the presence of magnesium ions [22,36] Specifically, mRNA integrity decreases at a higher rate at pH < 5.0 (citrate buffer) or at pH ≥ 8.0 in the case of HEPES, Tris and Sodium phosphate buffer [42]. Vaccines are generally formulated at a neutral pH (around 7.0–7.4) to preserve the mRNA efficacy (Table 3). For example, the approved Moderna and Pfizer/BioNTech COVID-19 vaccines have their pH controlled between 7 and 8 [58]. The early formulation trials of CureVac suggested that the product has the potential to maintain stability at a slightly acidic to neutral pH (6.5–7.5).

#### 4.2.4. Moisture

Humidity can promote hydrolytic reactions in dried lipid matrices, and residual moisture may accelerate both mRNA and lipid degradation. Therefore, strict control of residual water and storage humidity is critical for experimental lyophilized mRNA-LNP vaccines. However, to date, there are no licensed lyophilized mRNA vaccines, and these considerations are based on pre-clinical and early-development formulations rather than approved products [33]. The significant reduction in moisture content effectively inhibits the rate of hydrolysis of mRNA, which is considered the primary factor contributing to the instability of mRNA vaccines [60]. Therefore, vaccines are often sealed in moisture-proof packaging. All recently approved mRNA vaccines are typically stored in liquid form at low temperatures.

### 4.3. LNP Degradation Mechanisms

LNP degradation can occur through oxidation, hydrolysis, and related physical and chemical changes in the ionizable lipids and helper lipids, which together reduce particle integrity and transfection activity [54]. Reactive degradants from ionizable lipids can also form covalent bonds with mRNA, directly impairing translation. In addition, storage stress can induce lipid peroxidation and structural instability, making it more vulnerable to aggregation or loss of encapsulation [45]. These pathways are important because they can lead to reduced effectiveness even when the nanoparticles still appear physically intact. Overall, LNP stability depends on formulation conditions such as temperature, pH, and lipid composition [11].

#### 4.3.1. Chemical Degradation of LNPs

The chemical deterioration of mRNA vaccines can also arise from the processes of hydrolysis and oxidation of lipid nanoparticles (LNPs) (Figure 3). Both degradation routes affect LNP integrity, encapsulation efficiency, colloidal stability, and endosomal escape capability, which collectively impair cytosolic mRNA delivery and expression.

##### LNP Hydrolysis 

Ester bonds within helper phospholipids (e.g., DSPC) and ionizable cationic lipids (e.g., ALC-0315, SM-102) induce LNPs to undergo hydrolytic degradation in aqueous environments. The mechanism involves nucleophilic attack by water (or hydroxide) on the electrophilic carbonyl carbon of the ester. This forms a tetrahedral intermediate that collapses to release free fatty acids or monoacylglycerols with modified headgroups [61]. Hydrolysis rates are accelerated by elevated temperature, alkaline pH, and enzymatic catalysis (e.g., lipases or phospholipases) [62]. Non-enzymatic base hydrolysis predominates in LNP formulations, with half-lives ranging from days to months depending on lipid structure and conditions. Steric hindrance around the ester and lipid ionization state modulate susceptibility [34]. For instance, protonated ionizable lipids may shield esters, while oxidation byproducts can further promote hydrolysis. Degradation products like free fatty acids disrupt lipid packing, increase membrane fluidity, and alter surface charge. These results highlight enhanced mRNA leakage, particle fusion or aggregation, loss of encapsulation efficiency, and colloidal instability during storage or distribution [11].

Packer et al. showed that ionizable lipid oxidation accelerates hydrolysis, resulting in loss of mRNA activity within the LNPs. UHPLC analysis of stored COVID-19 mRNA-LNP revealed significant DSPC and cholesterol degradation in larger particles (120–150 nm) after 6 months at 4 °C, correlating with potency decline, whereas smaller (80–100 nm) LNPs maintained integrity [45]. Strategies like lyophilization or relatively small lipid analogs mitigate these issues by limiting water access.

##### LNP Oxidation

The major degradation mechanism for lipids in LNPs is oxidation, which is triggered by exposure to light, molecular oxygen, residual metal ions (e.g., Fe^3+^, Cu^2+^), and elevated temperatures, which generate reactive oxygen species (ROS) capable of damaging the lipids [63]. Carboxylic ester bonds in helper phospholipids like DSPC and ionizable cationic lipids (e.g., ALC-0315, SM-102) are particularly exposed. The primary targets are the polyunsaturated tails of helper lipids and cholesterol, which undergo oxidation to form hydroperoxides, malondialdehyde, 4-hydroxynonenal, and other reactive aldehydes [33]. These oxidized species destabilize LNP membranes by altering packing and increasing permeability.

#### 4.3.2. Physical Instability of LNPs

LNPs exhibit physical instability, undergoing aggregation, fusion, or mRNA leakage during prolonged storage, repeated freeze–thaw cycles, or mechanical agitation (e.g., shipping vibrations). These elevate hydrodynamic diameter, polydispersity index (PDI), and expose mRNA to the aqueous bulk phase where it undergoes rapid hydrolysis and RNase degradation [64]. Ice crystal formation during freezing imposes osmotic stress and mechanical deformation on lipid bilayers, driving particle fusion or mRNA release upon thawing [65]. The unprotected LNPs in PBS shows near-complete loss of delivery efficacy after just two freeze–thaw cycles [66]. High temperatures (e.g., ≥25 °C) further destabilize structural integrity by inducing gel-to-liquid crystalline phase transitions or lipid matrix reorganization. Also, the residual ethanol (>1% *v*/*v*) can increase LNP permeability [31,67]. Studies confirm that without stabilizers like sucrose or trehalose, freeze–thaw leads to PDI > 0.3 and encapsulation efficiency drops >20%, while optimized lyophilized formulations maintain size at <150 nm and potency for months at 4 °C [42,68]. These interconnected instabilities highlight the need for novel cryoprotectants to enable less stringent cold-chain logistics without compromising mRNA-LNP efficacy [69].

### 4.4. LNP Degradation: Key Factors

#### 4.4.1. Temperature

Storage temperature critically governs LNP stability, with elevated temperatures accelerating lipid hydrolysis/oxidation and physical aggregation, while lower temperatures markedly slow these processes and extend shelf life. In liquid mRNA-LNP formulations, higher temperatures accelerate ester bond cleavage in 1,2-distearoyl-sn-glycero-3-phosphocholine (DSPC) and ionizable lipids and peroxidation of unsaturated tails [4,7,53]. Ultra-low cryopreservation with 5–10% cryoprotectants like sucrose or trehalose vitrifies the aqueous matrix to preserve encapsulation efficiency and transfection activity for years [11]. Sato et al. showed that at 25 °C, acidic buffers accelerate ester hydrolysis over 8 weeks, while 4 °C storage maintains gene expression for similar periods in optimized ionizable lipid LNPs [11].

#### 4.4.2. pH, pKa and Buffer Composition

Buffer pH modulates LNP stability by governing ester hydrolysis rates in ionizable lipids (e.g., MC3, ALC-type, TOT-28), membrane ionization, and lipid packing. Sato et al. demonstrated that at 25 °C, lower buffer pH (e.g., 5.0 vs. 7.0) accelerates TOT-28 ester hydrolysis due to increased membrane hydration from protonated headgroups, whereas at 4 °C, the trend reverses slightly owing to pH-dependent changes in microviscosity that alter the hydrophilic microenvironment [11,52]. Biophysical characterization (SAXS, DLS) shows pH-induced phase transitions in LNPs, from inverse hexagonal (HII) to lamellar or micellar structures around pKa (~6), that influence colloidal stability [11]. The acidic pH promotes positive charge and aggregation propensity while neutral pH favours stable packing. For MC3-LNP, pH 5 triggers Fd3m cubic-to-hexagonal shifts correlating with endosomal escape efficiency, but storage at imbalanced pHs accelerates degradation. For instance, citrate buffers (pH 4–6) outperform phosphate by minimizing oxidation while supporting ionization [34].

The pKa of the ionizable lipid is commonly measured in assembled LNPs using the TNS fluorescence assay. This is considered a critical quality attribute responsible for colloidal stability, lipid phase behaviour, and delivery function. LNPs should remain near-neutral at physiological pH to limit aggregation in the endosome and promote the lamellar-to-inverse-hexagonal transition required for mRNA release. pKa values of approximately 6.2–6.5 are often associated with efficient hepatic delivery, whereas slightly higher values of ~6.6–6.9 may be favourable for intramuscular immunogenicity [70]. ALC-0315 in Comirnaty has been reported to have a pKa near 6.09, while SM-102 in Spikevax lies closer to the intramuscularly favourable range [11,57]. Importantly, the apparent pKa is not a fixed property during storage. Chemical degradation of the ionizable lipid, including N-oxide formation or ester cleavage, can alter headgroup charge behaviour and shift the particle’s apparent pKa [56,70]. This provides a mechanistic link between lipid chemical degradation and loss of biological activity.

#### 4.4.3. Moisture

Water inside LNPs is also a key player in the hydrolysis of both lipidic components and mRNAs [11]. Moisture serves as a primary driver of LNP degradation by directly facilitating hydrolytic cleavage and accelerating chemical reactions within lipid nanoparticles (LNPs) [68]. In liquid formulations stored at ultra-cold temperatures, residual water molecules within the LNP core enable the 2′-OH nucleophilic attack on phosphate groups, even under frozen conditions over time. Lyophilization (freeze-drying) improves stability by reducing moisture content, inhibiting hydrolysis, and enabling refrigerated storage (2–8 °C) for weeks to months without significant loss of encapsulation efficiency. High humidity during storage or transport can reintroduce moisture, triggering moisture-dependent degradation [12].

#### 4.4.4. Composition and Quality of Lipids

The chemical structure and purity of ionizable lipids (e.g., ALC-0315, MC3, SM-102), phospholipids (DSPC), cholesterol, and PEG-lipids (DMG-PEG) determine the LNPs’ susceptibility to hydrolysis and oxidation. Impurities like peroxides, N-oxides, aldehydes, or residual solvents (e.g., ethanol, DCM) from synthesis and manufacturing greatly accelerate breakdown and mRNA adduct formation [61]. Unsaturated tails in ionizable lipids and cholesterol are prone to peroxidation, yielding reactive aldehydes (e.g., malondialdehyde) that covalently modify mRNA nucleobases or phosphates. The N-oxides hydrolyze to amines and aldehydes under storage conditions, reducing translational potency. Birdsall et al. quantified aldehyde levels > 0.05% in 30% of ALC-0315 batches via DNPH derivatization/LC-MS, correlating degradation with the presence of impurities [62]. Packer et al. also showed N-oxide impurities in lipids cause mRNA deactivation via adduction [45]. A single adduct on a long transcript can abolish full-length translation. Standard batch maintained N-oxide at < 0.03% and aldehydes at < 0.015% through purification, underscoring regulatory needs for >99% purity to ensure LNP stability [71].

#### 4.4.5. Mechanical Stress

Shaking or mechanical agitation during handling, manufacturing, or transport introduces liquid–air interfaces that damage LNP lipid bilayers. Short exposures (e.g., 30 min at 100 rpm vertical shaking) cause minor size changes. In contrast, prolonged stress (240 min) induces PEG-lipid desorption, particle fusion/merging with a >4-fold size increase (e.g., 80–150 nm), increase in PDI and unencapsulated mRNA release, which were confirmed by cryo-EM, NTA, and RNase-sensitive thionine staining [48,65]. Also, Ruppl et al. showed that orbital shaking (300–600 rpm) damages nucleic acid encapsulation and reduces potency in eGFP-LNP assays. NMR also showed changes in lipid movement, highlighting the importance of reducing vibration during transport and using stabilizers to maintain stability [72].

#### 4.4.6. Freeze–Thaw Cycles

Repeated freeze–thaw cycles impose osmotic stress, ice crystal formation, and mechanical deformation on LNPs, driving irreversible aggregation, particle fusion, size enlargement, and loss of encapsulation efficiency that severely impair mRNA-LNP functionality [73]. Without cryoprotectants, even 2–3 freeze–thaw cycles reduce mRNA-LNP potency by more than 50% due to LNP rupture and mRNA leakage, as ice excludes solutes and concentrates salts/lipids in unfrozen pockets, promoting phase separation [7,74]. Sucrose (5–10%), trehalose, or betaine at 1:1–1:5 LNP weight ratios vitrify the matrix, maintain size at <150 nm, PDI < 0.2, and encapsulation >85% after ≥6 cycles by forming a glassy state that limits molecular mobility and ice damage. Further, PBS formulations fail after 5 cycles [7,69,74].

## 5. Stabilization Strategies to Overcome Instability

Previously, we covered how mRNA-LNP formulations lose stability and potency through chemical, physical, and environmental pathways. These challenges vary in terms of mitigation strategy: hydrolysis needs water removal or pH control; oxidation involves metal chelation and radical scavenging; and physical instability requires colloidal protection. The shift from ultra-cold to relaxed storage shows that these issues can be addressed through rational formulation. This section links each degradation mechanism to its corresponding mitigation strategy.

### 5.1. Excipient and Buffer Optimization

Buffer and excipient selection plays a key role in protecting against hydrolysis, oxidation, and freeze–thaw-induced structural damage. By controlling pH shifts, the aqueous environment can substantially influence mRNA-LNP stability. For example, the original BNT162b2 product used phosphate-buffered saline (PBS) and required dilution with saline before injection [75]. In late 2021, a Tris/tromethamine-sucrose formulation was approved by both the EMA and FDA, eliminating the dilution step and extending its frozen and refrigerated shelf life [76]. This change was physico-chemically important because phosphate buffers can undergo a pH shift during freezing, whereas Tris better resists this shift. This helps limit acid-catalyzed hydrolysis of both the mRNA backbone and ester-containing lipids during freeze–thaw transitions. Spikevax similarly uses a trometamol/acetate–sucrose cryoprotectant rather than PBS [70].

Sucrose, present at approximately 10% *w*/*v* in both Comirnaty and Spikevax, acts as a cryoprotectant by vitrifying the aqueous matrix and reducing ice crystal-induced disruption of the lipid bilayer (Table 4) [69,70]. Additional protection, for example, EDTA and citric acid, is used in Comirnaty. EDTA chelates trace divalent metals such as Fe^2+^ and Cu^2+^ that catalyze oxidation and backbone cleavage, while citrate helps suppress oxidative side reactions [77]. Experimentally, Reinhart et al. showed that C12-200- and SM-102 based mRNA–LNPs retained largely stable particle size, polydispersity, encapsulation efficiency, and mRNA content during nonfrozen storage. However, functional stability was formulation dependent, as C12-200 LNPs maintained EGFP expression for 11 weeks at both 2–8 and 25 °C, whereas SM-102 LNPs remained active at 2–8 °C but progressively lost transfection activity at 25 °C [14].

### 5.2. Lipid and Sequence Engineering

Because some degradation pathways originate from the intrinsic chemistry of the RNA and ionizable lipid, stabilization can also be built directly into the molecular design. Lipid engineering targets hydrolysis and oxidation, while mRNA sequence engineering reduces vulnerable regions within the transcript. Ester hydrolysis and lipid-tail peroxidation are major problems of many ionizable lipids, as discussed in Section 4.3. Therefore, next-generation lipid design is increasingly focusing on improving chemical robustness rather than relying solely on external formulation controls. Hashiba et al. demonstrated that piperidine-based ionizable lipids improved the thermostability of mRNA-LNPs, allowing them to retain gene expression and particle integrity under thermal stress [47]. Such chemically stable lipids may reduce dependence on ultra-cold storage by lowering the intrinsic rate of ester cleavage and oxidation.

Further, mRNA sequence engineering is a key strategy for improving the stability of therapeutic mRNAs. Codon optimization and balanced GC contents enhance translation efficiency and increase resistance to hydrolytic degradation. Also, advanced designed 5′ and 3′ untranslated regions (UTRs) and optimized poly(A) tails prolong intracellular half-life by promoting efficient ribosome recruitment and reducing susceptibility to cellular degradation pathways [78]. In addition, minimizing inhibitory secondary structures near the 5′ end and eliminating destabilizing sequence motifs, such as AU-rich elements, further improves mRNA stability [79]. Incorporation of modified nucleosides, particularly N1-methylpseudouridine, also reduces activation of innate immune response [80]. Collectively, these sequence engineering approaches increase the physicochemical stability, intracellular persistence, and translational efficiency of mRNA.

### 5.3. Solid-State Formulation: Lyophilization and Drying Approaches

Since water directly drives mRNA hydrolysis, removing bulk water is one of the most direct stabilization strategies. Solid-state formulations aim to slow chemical degradation while preserving LNP size, encapsulation, and biological activity after reconstitution. Lyophilized mRNA-LNP formulations stabilized with sucrose or trehalose have retained particle sizes below 150 nm, polydispersity indices below 0.2, and transfection activity for months at 2–8 °C in multiple independent studies (Table 4) [7,29]. Spray-freeze-drying has also been investigated to generate inhalable powder formulations while preserving mRNA integrity [13]. However, drying introduces its own stresses: freezing and dehydration can create osmotic and mechanical stress, leading to aggregation or mRNA leakage if cryoprotectant composition and ionizable lipid chemistry are not co-optimized. The authors in [81] addressed this limitation using a dual-function trehalose strategy, in which trehalose was incorporated both externally to vitrify the surrounding matrix and internally within the LNP core to stabilize mRNA through hydrogen bonding [82]. However, no licensed lyophilized mRNA vaccine is currently available at the time of writing, and these approaches remain in late development and are not approved product formats.

### 5.4. Process and Container Controls

Process and container controls are important for limiting physical disruption, photochemical damage, and impurity-driven degradation. Minimizing liquid–air interfaces and mechanical agitation help limit PEG-lipid desorption and bilayer damage, as discussed in Section 4.4.5 [44,60,66]. Amber or opaque primary containers, together with light-protective secondary packaging, reduce photo-oxidation risk (Section 4.2.2). In addition, strict control of residual solvents, peroxides, and aldehyde impurities in raw ionizable lipids limits the formation of reactive degradants that can drive mRNA adduction (Section 4.4.4) [55,57,65].

## 6. Analytical Methods for Assessing mRNA-LNP Integrity and Quality

A central challenge in evaluating mRNA-LNP stability is that physicochemical integrity and biological potency may change [55]. Therefore, mRNA-LNP characterization requires multi-attribute testing that captures mRNA integrity, encapsulation, particle structure, and biological potency [83]. This section summarizes the principal methods used to assess these quality attributes, with emphasis on approaches reported in industrial practice, regulatory filings, and peer-reviewed studies (Table 5).

### 6.1. mRNA Integrity and Purity

The most widely used methods for measuring mRNA size are ion-pair reversed-phase high-performance liquid chromatography (IP-RP-HPLC) and capillary gel electrophoresis (CGE). Both separate intact transcripts from degradation fragments, but they rely on different principles [3,84]. IP-RP-HPLC separates species based on hydrophobicity in the presence of ion-pairing reagents, whereas CGE separates RNA by size through a sieving gel matrix [51]. Mantri et al. found that different methods can give different estimates of mRNA degradation. In some cases, the results differed by up to 50%. The difference depended on the mRNA length, storage temperature, and whether the mRNA was inside LNPs [77]. IP-RP-HPLC is also useful for detecting mRNA-lipid adducts. Packer et al. showed that adducted mRNA in stored COVID-19 mRNA-LNP formulations elute due to increased hydrophobicity from covalently attached lipid portions [55]. Mass spectrometry confirmed that these adducts occur mainly on cytosine residues and arise from reactive aldehydes generated by ionizable-lipid degradation [85]. For higher-throughput testing, multi-capillary CGE platforms such as the SCIEX BioPhase 8800 enable parallel analysis of RNA purity and integrity after extraction from LNP samples [86].

### 6.2. mRNA Quantification and Encapsulation Efficiency

Encapsulation efficiency determines how much mRNA is protected inside the LNP rather than exposed to the surrounding medium. This attribute is essential because unencapsulated mRNA is more susceptible to degradation and is less effective at delivery. Encapsulation efficiency is commonly measured using the RiboGreen fluorescence assay. In this method, RiboGreen detects unencapsulated mRNA in the absence of detergent and total mRNA after LNP disruption with Triton X-100. The ratio of free to total RNA is then used to calculate percent encapsulation [83]. Although rapid and scalable, RiboGreen does not distinguish intact mRNA from fragmented RNA. Therefore, total mRNA and encapsulation measurements are often complemented by orthogonal approaches such as LC-MS/MS or analytical ultracentrifugation (AUC), which can also provide information on particle density and sedimentation heterogeneity [83,87].

### 6.3. Particle Size, Polydispersity, and Morphology

Particle size and morphology provide information on the physical stability of the LNP carrier. These measurements are especially important for detecting aggregation, fusion, leakage, or structural changes. Dynamic light scattering (DLS) remains the standard method for measuring hydrodynamic diameter and polydispersity index (PDI) and is commonly used as a release assay for mRNA–LNP products. Nanoparticle tracking analysis (NTA) complements DLS by providing number-weighted size distributions and particle concentration. Hermosilla et al. used comparative particulate analysis of in-use Comirnaty and Spikevax samples, demonstrating the value of these methods for monitoring real-world product quality [16]. For deeper structural analysis, cryogenic transmission electron microscopy (cryo-TEM) and small-angle X-ray scattering (SAXS) can reveal LNP morphology and internal organization, including lamellar and inverse hexagonal phases. These structural states are relevant to both stability and endosomal escape, as discussed in Section 4.4.2. Asymmetric flow field-flow fractionation coupled to multi-angle light scattering (AF4-MALS) and AUC provide additional orthogonal assessments of size heterogeneity and particle density, allowing for detection of subpopulations that may be undetected by DLS [81].

### 6.4. Lipid Degradation, Impurities, and Apparent pKa

Lipid degradation can compromise both the carrier and the mRNA cargo. Therefore, analytical methods must monitor not only lipid identity and purity but also reactive impurities that may trigger mRNA adduction or potency loss. Liquid chromatography–mass spectrometry (LC-MS) is the primary tool for lipid identity and degradation analysis. Birdsall et al. used DNPH derivatization coupled to LC-MS to quantify aldehyde impurities in ALC-0315 raw material batches, detecting levels above 0.05% in approximately 30% of tested lots and linking impurity burden to subsequent mRNA degradation [57]. Similarly, Packer et al. used LC-MS to identify cytosine adducts formed when reactive lipid degradants interacted with encapsulated mRNA [88]. These studies establish LC-MS as a key method for detecting impurity-driven degradation pathways. The apparent pKa of the ionizable lipid within the assembled LNP is another critical quality attribute because it influences both colloidal stability and endosomal escape. It is commonly measured using the TNS fluorescence assay, in which the anionic probe binds positively charged lipid surfaces in a pH-dependent manner. The inflection points of the fluorescence–pH curve define the apparent pKa [82]. TNS-derived pKa measurements have supported empirical design ranges for mRNA-LNP activity, including approximately 6.2–6.5 for hepatic delivery and 6.6–6.9 for intramuscular immunogenicity [88].

### 6.5. Biological Potency

Biological potency is the final functional test of whether the formulation still performs its intended role. Even when physical and chemical assays appear acceptable, potency assays are needed to confirm that the mRNA can be delivered and translated. In vitro potency is typically assessed using reporter-gene mRNA, such as enhanced green fluorescent protein or firefly luciferase, delivered to cultured cells. Fluorescence or luminescence then serves as a surrogate for protein expression. In vivo potency is evaluated through immunogenicity studies, including neutralizing antibody titers and T-cell responses [55,80]. Potency assays remain less standardized than physicochemical methods because results depend on cell type, assay format, and animal model. However, they are indispensable. Packer et al. showed that mRNA-lipid adducts invisible to size-based assays can reduce translational output by more than 50%, making biological potency the definitive measure of product functionality [55].

### 6.6. The Need for Orthogonal Characterization

Because each method captures only one aspect of stability, mRNA-LNP quality assessment must be multi-dimensional. A formulation may appear stable in one assay while showing degradation in another. For example, DLS may show unchanged particle size even when IP-RP-HPLC detects mRNA fragmentation, LC-MS reveals mRNA–lipid adducts, or TNS detects an apparent pKa shift. Any of these defects can reduce potency without obvious changes in particle diameter. Therefore, a minimal stability-indicating panel should include at least one RNA integrity method, such as IP-RP-HPLC or CGE, one quantification and encapsulation method, such as RiboGreen, one particle-sizing method, such as DLS, and one biological potency assay. LC-MS should be added when lipid degradation, reactive impurities, or adduct formation are suspected [55,77,80]. Standardizing such orthogonal panels across manufacturers and regulatory jurisdictions remains an important unmet need for the mRNA vaccine field.

## 7. Conclusions and Future Perspectives

The rapid clinical success of mRNA-LNP products has demonstrated the groundbreaking potential of this platform. However, stability remains one of the greatest barriers to its broader application. As discussed throughout this review, the stability of mRNA-LNP formulations is governed by a complex interplay of physicochemical degradation pathways affecting both the mRNA cargo and the lipid nanoparticle carrier. Temperature fluctuations, pH variations, oxidative stress, moisture, mechanical stress, and enzymatic degradation collectively affect molecular integrity, encapsulation efficiency, and biological activity. Importantly, these degradation mechanisms are highly interconnected rather than independent, meaning that degradation of one component often accelerates deterioration of the other. Consequently, improving product stability requires a broader understanding that integrates RNA chemistry, lipid design, nanoparticle architecture, manufacturing processes, analytical characterization, and storage conditions.

Despite considerable advances in formulation science, current stabilization strategies primarily address the physical indicators of instability rather than the underlying chemical degradation mechanisms. Cryoprotectants, lyoprotectants, optimized buffer systems, and lyophilization have significantly improved storage performance by reducing aggregation, fusion, and leakage of lipid nanoparticles. However, these approaches provide limited protection against phosphodiester backbone hydrolysis, ionizable lipid hydrolysis, lipid oxidation, or the formation of reactive degradation products that can chemically modify mRNA. Future progress will therefore depend on preventing chemical degradation at its source rather than simply slowing its physical deterioration.

Another major unresolved challenge is the design of next-generation ionizable lipids that simultaneously provide efficient intracellular delivery and improved chemical stability. Most clinically approved ionizable lipids were developed to maximize endosomal escape and biodegradability, but the ester bonds and oxidation-sensitive functional groups incorporated into these molecules also make them susceptible to hydrolysis and oxidation during storage. Future lipid engineering should therefore prioritize chemically robust molecular architectures with improved resistance to oxidation and hydrolysis. Advances in computational chemistry, molecular dynamics simulations, lipid discovery, and high-throughput screening are expected to accelerate the rational development of more stable lipid materials.

In addition to formulation challenges, greater attention should be directed toward the influence of manufacturing processes on long-term product stability. Degradation can begin during in vitro transcription, purification, nanoparticle assembly, sterile filtration, fill-finish operations, freezing, or transportation, where factors such as residual solvents, dissolved oxygen, trace metal contaminants, mixing conditions, and freeze–thaw cycles may significantly influence product quality. Understanding how these upstream variables contribute to downstream degradation will be critical for establishing robust manufacturing processes and ensuring batch-to-batch consistency.

A further limitation in the field is the absence of standardized analytical methodologies capable of reliably linking physicochemical stability with biological performance. Current quality control primarily relies on measurements such as particle size, polydispersity index, encapsulation efficiency, and RNA integrity. While these parameters provide valuable information, they frequently fail to predict in vivo potency. Critical quality attributes such as ionizable lipid oxidation, lipid hydrolysis, lipid–RNA interactions, and endosomal escape efficiency are not routinely monitored, making comparisons between studies difficult and complicating regulatory evaluation. The development of consistent analytical standards and biologically relevant potency assays will therefore be essential for improving comparability across laboratories and facilitating regulatory approval of future mRNA products.

In addition, current stability studies also remain largely empirical and product-specific, limiting their usefulness for predicting long-term performance across diverse formulations. Most shelf-life assessments rely on extensive real-time stability testing, which is both time-consuming and resource-intensive. Future research should move toward predictive, mechanism-based stability modeling by integrating degradation kinetics, machine learning, and Quality-by-Design principles. Such approaches could enable rational formulation optimization, stability prediction, and proactive process control throughout manufacturing, storage, transportation, and distribution.

Overcoming mRNA-LNP instability requires coordinated advances across formulation science, lipid chemistry, analytical technology, manufacturing engineering, computational modeling, and regulatory science. The evolution from strict ultra-low-temperature storage requirements of licensed mRNA vaccines to increasingly flexible storage conditions demonstrates that meaningful improvements are achievable through rational formulation design. Addressing these unresolved challenges will not only enhance the commercial viability of mRNA therapeutics but will also expand global access to vaccines and other nucleic acid medicines. This helps strengthen preparedness for future infectious disease outbreaks and enables the broader application of mRNA technology across numerous therapeutic areas.

## Figures and Tables

**Table 1:** Factors affecting mRNA-LNP stability, with mechanism, impact, and mitigation strategies.

Factor	Underlying Mechanism	Impact on Product Quality	Mitigation Strategies
Temperature	Arrhenius-accelerated backbone hydrolysis; lipid oxidation; phase transitions	Loss of full-length mRNA; aggregation; potency loss	Frozen/cold chain; lyophilization; thermostable lipids
pH	Extreme pH catalyzes hydrolysis; shifts lipid ionization	Fragmentation; altered charge/aggregation	Buffer at pH 7.0–7.4; citrate vs phosphate choice
Light (UV/Vis)	ROS generation; photo-oxidation of bases/lipids	Strand breaks, cross-links, reduced potency	Amber/opaque vials; light protection
Oxidation	Lipid peroxidation → reactive aldehydes → mRNA adducts; metal-catalyzed ROS production	Adducted, non-translatable mRNA; membrane damage	Antioxidants; EDTA; inert headspace; lipid purity
Freeze–thaw	Ice formation; osmotic/mechanical stress	Fusion, leakage, >50% potency loss	Sucrose/trehalose 5–10%; controlled freezing
Moisture	Residual water enables hydrolysis of mRNA and ester lipids	Slow chemical degradation even when frozen	Lyophilization; sealed low-humidity packaging
Mechanical/shear	Liquid–air interface; PEG-lipid desorption; bilayer defects	Size increase, PDI rise, unencapsulated mRNA	Minimize agitation/vibration; surfactant stabilizers
Impurities	Peroxides, N-oxides, aldehydes, residual solvent	mRNA adduction; potency loss	>99% lipid purity; LC-MS impurity monitoring
Enzymes (RNase)	Exo-/endonucleolytic cleavage of exposed mRNA	Rapid loss of leaked mRNA	RNase-free process; encapsulation
Aggregation	Colloidal destabilization; fusion	Reduced delivery efficiency and efficacy	Steric (PEG) stabilization; ionic/pH control

**Table 3:** Vaccines with their optimal storage pH [10,34,59].

Vaccine Type	Vaccine Example	Optimal Storage pH Range
mRNA Vaccines	Pfizer-BioNTech (Comirnaty)	7.0–7.4
	Moderna (Spikevax)	7.0–7.4
	CureVac (CVnCoV)	6.5–7.5

**Table 4:** Degradation pathway, stabilization strategy, and supporting evidence.

Degradation Pathway	Stabilization Strategy	Supporting Evidence
Backbone hydrolysis	Water removal; buffer optimization; metal chelation	Lyophilization; Tris/sucrose, EDTA formulation [69,71]
Oxidation of mRNA adducts	Antioxidants; chelators; lipid purity control	EDTA; aldehyde control in ALC-0315 batches [57,71]
Lipid ester hydrolysis and peroxidation	Chemically robust ionizable lipids	Piperidine-based lipids vs. MC3 [47]
Freeze–thaw and physical instability	Cryoprotectant sugars; surfactant–sugar pairs	~10% sucrose; sucrose–P188 [14,69,70]

**Table 5:** Summary of analytical methods for mRNA-LNP characterization.

Quality Attribute	Analytical Method(s)
mRNA integrity and purity	IP-RP-HPLC; CGE (BioPhase 8800; Fragment Analyzer)
mRNA–lipid adducts	IP-RP-HPLC coupled to LC-MS
Encapsulation efficiency and total RNA	RiboGreen fluorescence (± Triton X-100); LC-MS/MS; AUC
Particle size and PDI	DLS; MADLS; NTA
Morphology and internal phase	Cryo-TEM; SAXS; AF4-MALS
Lipid impurities and aldehydes	DNPH derivatization–LC-MS; LC-MS/MS
Apparent pKa	TNS fluorescence assay
Biological potency	In vitro reporter transfection (eGFP, Fluc); in vivo immunogenicity

## Data Availability

The original contributions presented in the study are included in the article; further inquiries can be directed to the corresponding author.
